# Acoustic feedback enables safe and reliable carboplatin delivery across the blood-brain barrier with a clinical focused ultrasound system and improves survival in a rat glioma model

**DOI:** 10.7150/thno.35892

**Published:** 2019-08-14

**Authors:** Nathan McDannold, Yongzhi Zhang, Jeffrey G. Supko, Chanikarn Power, Tao Sun, Chengueng Peng, Natalia Vykhodtseva, Alexandra J Golby, David A. Reardon

**Affiliations:** 1 Department of Radiology, Brigham and Women's Hospital, Harvard Medical School, Boston, MA; 2 Department of Medicine, Massachusetts General Hospital, Harvard Medical School, Boston, MA; 3 Department of Neurosurgery, Brigham and Women's Hospital, Harvard Medical School, Boston, MA; 4 Electrical Engineering, Boston University, Boston, MA; 5 Department of Medical Oncology, Dana-Farber Cancer Institute; Department of Medicine, Brigham and Women's Hospital; Harvard Medical School, Boston, MA

**Keywords:** Ultrasound, blood-brain barrier, brain tumor, chemotherapy

## Abstract

The blood-brain barrier (BBB) restricts delivery of most chemotherapy agents to brain tumors. Here, we investigated a clinical focused ultrasound (FUS) device to disrupt the BBB in rats and enhance carboplatin delivery to the brain using the F98 glioma model.

**Methods**: In each rat, 2-3 volumetric sonications (5 ms bursts at 1.1 Hz for 75s) targeted 18-27 locations in one hemisphere. Sonication was combined with Definity microbubbles (10 µl/kg) and followed by intravenous carboplatin (50 mg/kg). Closed-loop feedback control was performed based on acoustic emissions analysis.

**Results**: Safety and reliability were established in healthy rats after three sessions with carboplatin; BBB disruption was induced in every target without significant damage evident in MRI or histology. In tumor-bearing rats, concentrations of MRI contrast agent (Gadavist) were 1.7 and 3.3 times higher in the tumor center and margin, respectively, than non-sonicated tumors (P<0.001). Tissue-to-plasma ratios of intact carboplatin concentrations were increased by 7.3 and 2.9 times in brain and tumor respectively, at one hour after FUS and 4.2 and 2.4 times at four hours. Tumor volume doubling time in rats receiving FUS and carboplatin increased by 96% and 126% compared to rats that received carboplatin alone and non-sonicated controls, respectively (P<0.05); corresponding increases in median survival were 48% and 66% (P<0.01).

**Conclusion**: Overall, this work demonstrates that actively-controlled BBB disruption with a clinical device can enhance carboplatin delivery without neurotoxicity at level that reduces tumor growth and improves survival in an aggressive and infiltrative rat glioma model.

## Introduction

The use of focused ultrasound (FUS) and microbubbles to disrupt the blood-brain barrier (BBB) is a promising noninvasive nonthermal method to enable or enhance drug delivery to the central nervous system 1. This technique, which has been studied in numerous preclinical studies 2-8 and early clinical trials 9-11, utilizes the mechanical interactions between the ultrasound field, the circulating microbubbles, and the microvasculature to induce a temporary opening of the BBB in a focal region, thereby enabling the passage of even large-molecule drugs that do not normally reach the brain. The barrier opens for several hours, with a closing time dependent on the size of the drug or tracer 12.

This approach can also increase the permeability of the partially-intact blood-tumor barrier (BTB) and enhance the delivery of chemotherapy to CNS tumors 2-4. Importantly, it can also deliver drugs to the surrounding brain tissue, where infiltrating tumor cells are protected by a fully-intact BBB. Thus, this approach is a promising method for improving drug therapies for brain tumor patients.

The chemotherapy agent carboplatin is used clinically to treat various extracranial tumors, and has shown modest effects in patients with recurrent malignant glioma 13-15. While it will perhaps extravasate in areas lacking an intact BTB, little or none crosses the intact BBB 16, leading to ineffective treatment of infiltrating tumor cells, and therefore subsequent recurrence. For this reason, researchers have investigated alternative delivery methods to enhance delivery of carboplatin to infiltrating glioma including convection-enhanced delivery (CED) 17 or BBB disruption via arterial injections of hyperosmotic mannitol 18, the bradykinin agonist RMP-7 19, or via a surgically-implanted ultrasound device 9. Transcranial FUS offers potential advantages over these approaches, as it is noninvasive, easily repeated, and can be tailored to a specific patient's tumor location and size. Further, the BBB disruption lasts for several hours, allowing sufficient time for drug to accumulate. It also might suppress drug clearance via suppression of the drug efflux pump P-glycoprotein 20.

The clinical transcranial MRI-guided focused ultrasound (TcMRgFUS) device that is being used for BBB disruption 10,11 use lower ultrasound frequencies than have been employed in most preclinical studies in small animals. Clinically, employing a low frequency has advantages in reducing skull-induced aberration, increasing the focal region volume, and in electronic beam steering when phased array transducers are used. However, the larger focal region produced by low-frequency transducers can make testing clinical devices challenging in small animal models. Reflections and standing waves within the skull cavity can lead to unpredictable hotspots in the resulting acoustic field 21. If the geometric gain of the transducer is not high enough, the size of the focal region will exceed the dimensions of the brain, leading to high acoustic intensities near the skull due to internal reflections.

The purpose of this work was to test the ability of a clinical TcMRgFUS system that operates at a low frequency to reliably disrupt the BBB in rats and deliver carboplatin to the healthy brain without clinically-significant neurotoxicity, and to determine whether enhanced carboplatin delivery can improve survival in an aggressive glioma model. Further, a closed-loop feedback system based on recordings of acoustic emissions was integrated into the device and evaluated. We show that the large geometric gain achieved with a hemispherical transducer results in FUS-induced BBB disruption that can be reliably and repeatedly used in rats to enhance carboplatin delivery and prolong survival.

## Results

**Figure 1:**
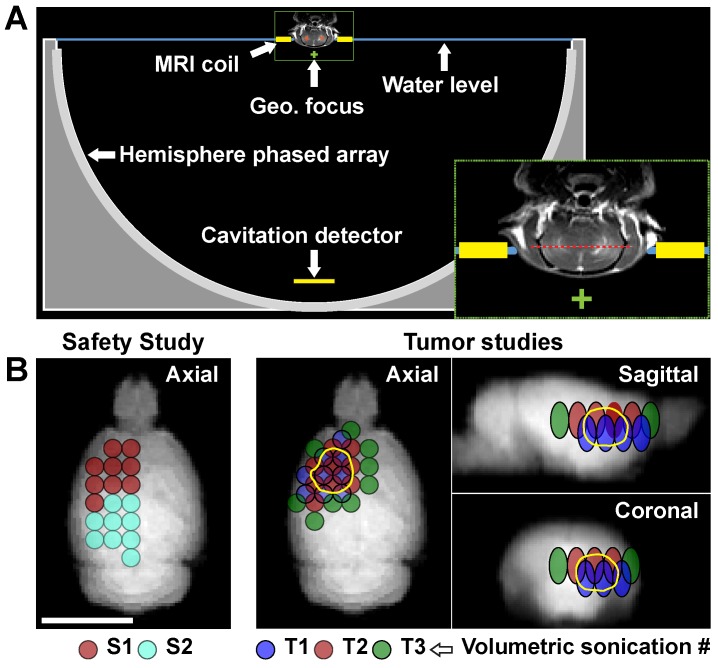
Experimental setup. **(A)** Diagram showing the 30 cm diameter hemispherical phased array with a coronal MRI of the rat head superimposed (to scale). The top of the head was partially submerged. A passive cavitation detector was placed near the bottom of the array. The geometric focus of the transducer (green +) was approximately one cm below the target axial plane in the rat brain (red dotted line in inset). **(B)** Locations of the individual sonication targets in each animal. The circles are the individual targets in the volumetric sonications; the different colors indicate the patterns used for the different volumetric sonication. In the safety study, two volumetric sonications were applied at one depth. In the tumor studies, the two or three volumetric sonications were applied at different depths to increase the volume of brain with BBB disruption. The third volumetric sonication (T3) was used on the second FUS+carboplatin treatment on day 29. The location of the tumor is indicated by the solid yellow line.

We first evaluated the safety of the BBB disruption and enhanced carboplatin delivery in the normal brain. We applied volumetric sonications that covered the entire right cerebrum in conjunction with carboplatin administration at 50 mg/kg. Diagrams showing the experimental setup and the locations of the targets are shown in **Figure 1**. Each volumetric sonication targeted nine locations via electronic beam steering. We first applied 30 s sonications without microbubbles followed by 75 s sonications with microbubbles. The acoustic power was controlled in real-time based on analysis of the acoustic emissions, and the tissue effects were evaluated with MRI and histology.

### Acoustic emissions and feedback control

**Figure 2:**
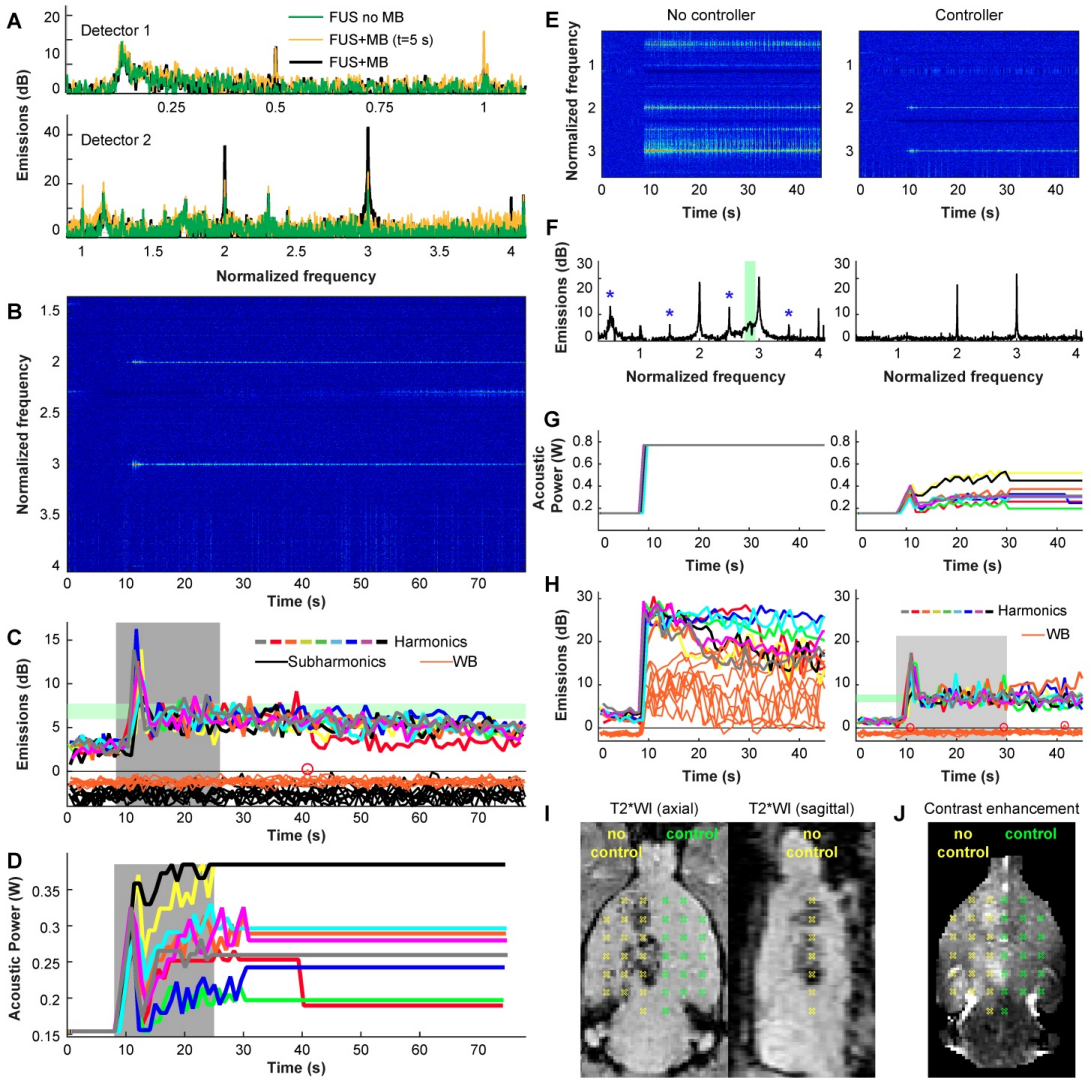
** (A-D)** Example acoustic emission obtained during a volumetric sonication with closed-loop feedback control. **(A)** Example spectra obtained with the two passive cavitation detectors during individual 5 ms bursts during a sonications with and without microbubbles (MB). With microbubbles, large and obvious enhancement at the second and third harmonics was observed, while subharmonic emission was unchanged. Data are shown in dB relative to the noise floor. **(B)** Spectrogram from detector 2. Here, the data is normalized to the data obtained between 1-8 s - before the microbubbles arrived in the brain; only harmonic emission is observed. **(C)** Magnitude of harmonic, subharmonic, and wideband (WB) emissions vs. time during the 9 locations targeted in a volumetric sonication. The magnitudes of the harmonic emissions were similar for the 9 locations, and all achieved the controller goal of 6-7.5 dB above the noise floor (blue region). Overshoot occurred during this sonication. Wideband and subharmonic emissions are shown relative to the threshold used to trigger a reduction of power; one pulse (circle) slightly exceeded the threshold for wideband emission. **(D)** Acoustic power vs. time for the 9 locations. During the control period (gray region), the power at each location was modified based on the magnitude of the harmonic emissions. After this time, the power level was fixed to the average value of all the bursts that were within the controller goal. In cases where subharmonic or wideband emissions were detected, the power level was reduced by 25% and fixed for the remainder of the sonication. **(E-J)** Demonstration of the controller's ability to maintain a safe and effective exposure level. In this rat, we set the maximum allowed power to double what was used in the other animals. We applied 2 volumetric sonications in each hemisphere; the controller was disabled for sonications in the right hemisphere. **(E-F)** Spectrograms and spectra (averaged here over 10-20s) reveal wideband emissions in the sensitive band of detector 2 (green region) when the controller was disabled. Subharmonic and ultraharmonic emissions were also seen (*). With the controller, the power was modulated **(G)** to achieve the desired harmonic emissions without wideband emissions **(H)**. The uncontrolled sonication resulted in large wideband emissions throughout the sonication **(H, left)**. Post-treatment MRI revealed significant hypointensities in T2*-weighted imaging (T2WI) without the controller **(I)**. With the controller, no abnormalities were observed in T2*-weighted MRI and homogenous contrast enhancement was observed in T1-weighted imaging **(J)**.

Example acoustic emissions and power control are shown in **Figure 2**A-D. These data were recorded during one volumetric sonication that targeted nine locations. For the first 8 s of the microbubble-enhanced sonication, the power level was fixed as the bubbles required this amount of time to reach the brain. Spectra obtained here were similar to those obtained without microbubbles (**Figure 2**A). After the microbubbles arrived, the only signals typically evident were the second and third harmonics (**Figure 2**B); the acoustic power was controlled based on their strength. The acoustic power level was modulated until the mean harmonic signal reached a target between 6-7.5 dB above the noise floor (**Figure 2**C). At 25 s the power was fixed to the average level that resulted in this target range for the remainder of the 75 s sonication to avoid overexposure as the microbubbles cleared from circulation (**Figure 2**D).

In every sonicated location, obvious and clear enhancement of harmonic emissions were observed, and the emissions reached the controller target although overshoot was observed in many cases. Such enhancement was not observed in sonications without microbubbles, demonstrating that the cavitation activity was due solely to the presence of the injected microbubbles. Similar results were evident in every sonication (Figure S1). Location 2 consistently required less power to achieve the same harmonic emissions. The mean power level at the end of the control period was 0.38 and 0.31 W for locations 1 and 2 respectively (corresponding peak pressure amplitude estimates: 161 and 144 kPa).

Increased subharmonic or wideband emissions, indicators of a risk of inertial cavitation and vessel damage, were observed with microbubbles in less than 10% of the sonicated locations and less than 1% of all bursts. The second location had a higher probability for wideband emissions (12% vs. 4.6%) and targets adjacent to the midline appeared to have a higher probability for subharmonic or wideband emissions (Figure S2). A summary of findings from the acoustic emissions recordings is shown in **Table S1**.

To demonstrate the ability of the controller to mainatain a safe exposure level, we doubled the maximum acoustic power level allowed by the controller and performed two volumetric sonications in the left hemisphere of a rat. We then disabled the controller and performed two volumetric sonications in the right hemisphere. The results of this experiment are shown in **Figure 2**E-J. With the controller disabled, large wideband emissions were observed in throughout the sonication (**Figure 2**E-H, left), and hypointense regions were observed in T2*-weighted imaging (**Figure 2**I). With the controller, only harmonics were observed, and the power was maintained at a safe level, despite our increasing the maximum power level allowed to an unsafe level. Uniform BBB disruption was observed in contrast-enhanced MRI (**Figure 2**J).

### BBB disruption after volumetric sonication

**Figure 3:**
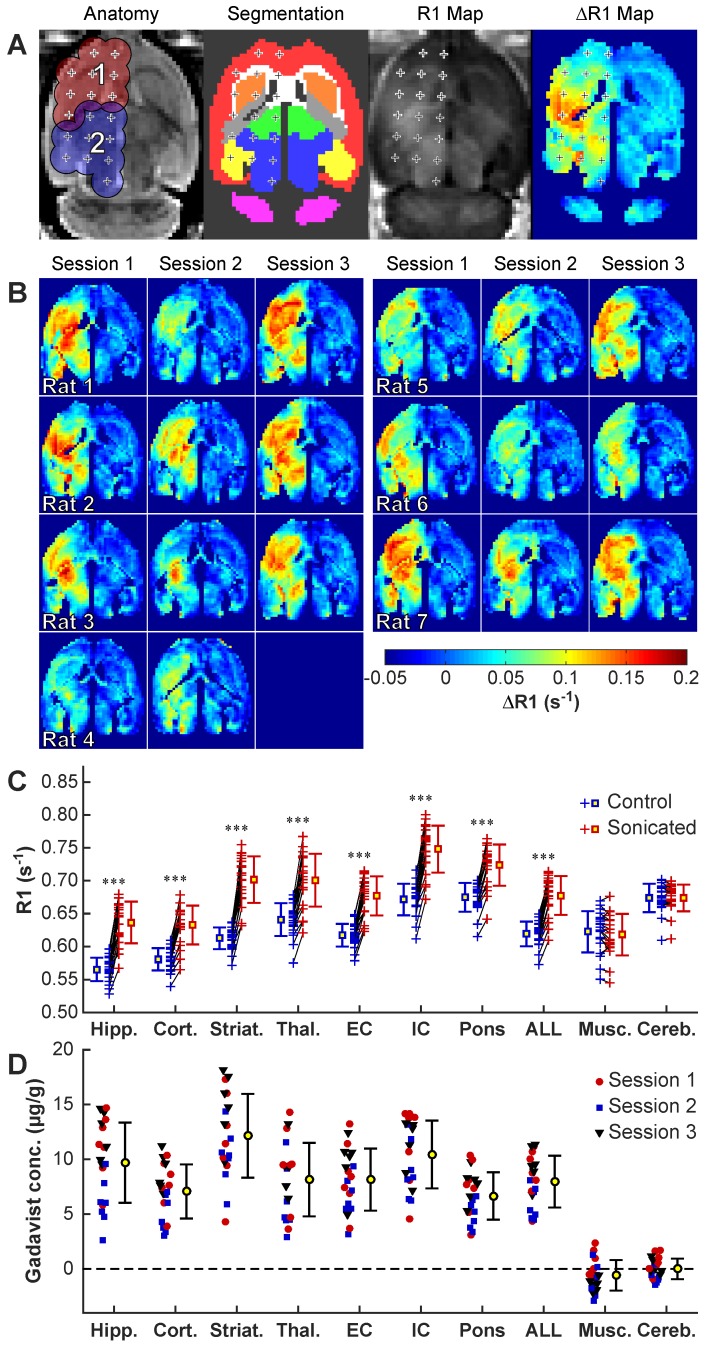
R1 mapping to visualize Gadavist delivery across the BBB over three sessions in 7 rats. **(A)** Anatomic images, segmentations of different brain structures, R1 maps and ΔR1 maps for one rat. The 18 sonication targets are indicated. The contours in A indicate a 1.5 mm radius around each target for the four volumetric sonications. These contours, along with the segmentations, were used to create ΔR1 maps showing differences between the sonicated and control hemisphere. **(B)** ΔR1 maps for 20 consecutive sessions in ten rats. While there was considerable variability, in every tissue structure within the sonicated volumes the mean R1 was significantly (P<0.001) higher when comparing the two hemispheres. **(C)** R1 changes for the different structures. Each + indicates the mean signal measured in that tissue structure in the sonicated hemisphere and in corresponding locations in the contralateral, non-sonicated hemisphere. Regions in neighboring muscle and in the cerebellum were not sonicated, and the differences in R1 were not significant. **(D)** Difference in Gadavist concentration between the sonicated and control hemispheres, estimated using the R1 measurements and the relaxivity of the contrast agent (4.44s^-1^· mM^-1^). *(Hipp: Hippocampus; Cort: Cortex; Striat: Striatum; Thal: Thalamus; EC: External capsule; IC: Internal capsule; Musc: muscle; Cereb: Cerebellum)*

We obtained maps of R1 relaxation to quantify the BBB disruption (**Figure 3**A). Gadavist delivery was evident in the R1 maps in every sonicated volume and session in the safety study (**Figure 3**B). A significant difference in R1 (P<0.001) between hemispheres was evident in every tissue structure that was included within ±1.5 mm of the sonication targets. Sagittal imaging revealed that the disruption was contained within the brain in most animals, although in some lateral targets the disruption reached the brain surface proximal to the transducer.

**Figure 3**C-D shows the differences in R1 values obtained in different tissue structures in the sonicated vs. non-sonicated hemispheres. We used these measurements to estimate differences in Gadavist concentrations compared to the control hemisphere, assuming a relaxivity for Gadavist of 4.44 mmol/s^-1^ 22. Significant changes (P<0.001) were observed in the sonicated regions but not in the non-sonicated cerebellum or in adjacent muscle. The highest enhancement in Gadavist delivery was observed in the striatum.

Serial T1-weighted imaging revealed spread of the contrast agent away from the sonication targets. Over the first ~10 min after injection of Gadavist, the enhancement pattern in the brain with T1-weighted imaging was spotty, and enhancement at individual sonication targets was evident; delayed imaging acquired ~25 min later showed a more homogeneous enhancement pattern (**Figure S3**D). Changes in R1 and signal enhancement in T1-weighted imaging for the two volumetric sonications are shown in **Figure S3**A-B. A good correlation (R²=0.69) was observed between the contrast enhancement in T1-weighted imaging and estimated Gadavist concentrations (**Figure S3**C). Signal enhancement in T1-weighted imaging and R1 relaxation rates were both slightly higher at both locations in male rats, but the difference was not significant (P=0.470, 0.137 respectively). R1 relaxation and signal enhancement changes in the T1-weighted imaging are further summarized in **Table S2**.

### No brain tissue damage evident after repeated BBB disruption and carboplatin delivery

Comparison of T2*-weighted MRI in the normal brain obtained before and after sonication revealed no obvious hypointensities that would indicate vascular damage or petechiae. In addition, T2-weighted imaging obtained 48h and weekly after each sonication session was normal in every animal (**Figure S4**).

**Figure 4:**
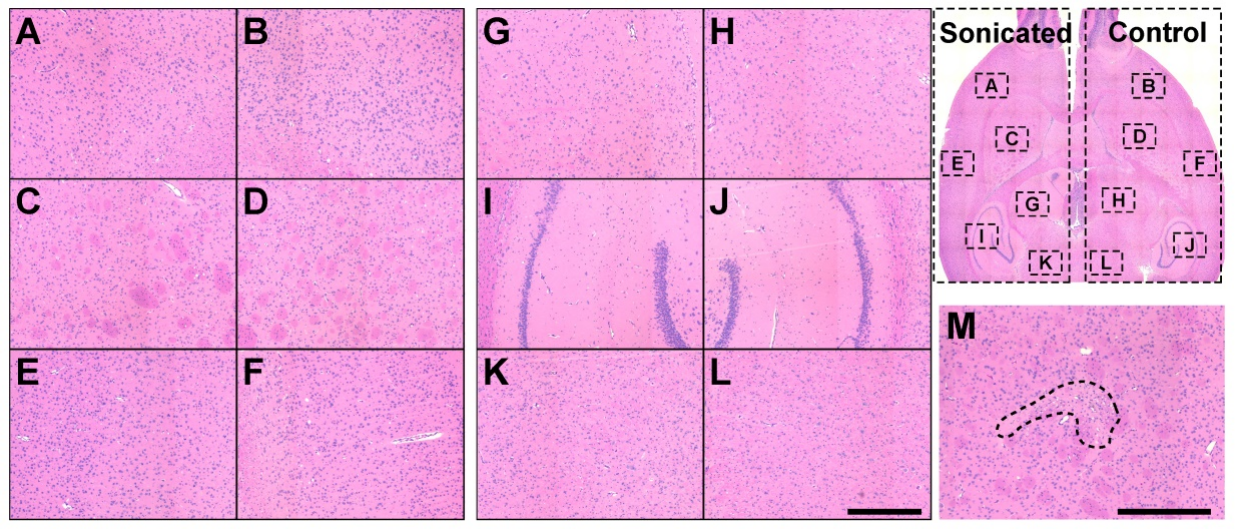
Safety study histology. **(A-L)**: Microphotographs of a typical H&E-stained section of a rat brain after three tri-weekly sessions of FUS-induced BBB disruption and carboplatin. The brain appeared normal. **(M)** A tiny scar found in the striatum in a different rat was the only evident possible damage that was found. *(Bar: 500 µm)*.

Two H&E-stained sections at a central plane were investigated in each rat for FUS- and/or carboplatin-induced neurotoxicity. The overwhelming majority of the sonicated regions appeared normal. Representative findings from one rat are shown in **Figure 4**(A-L). The only abnormality detected among the rats was a tiny (dimensions less than one mm) scar in the striatum in one animal (**Figure 4**M). This animal received two volumetric sonications at different depths.

### Blood-tumor barrier disruption and carboplatin concentrations in F98 glioma

**Figure 5:**
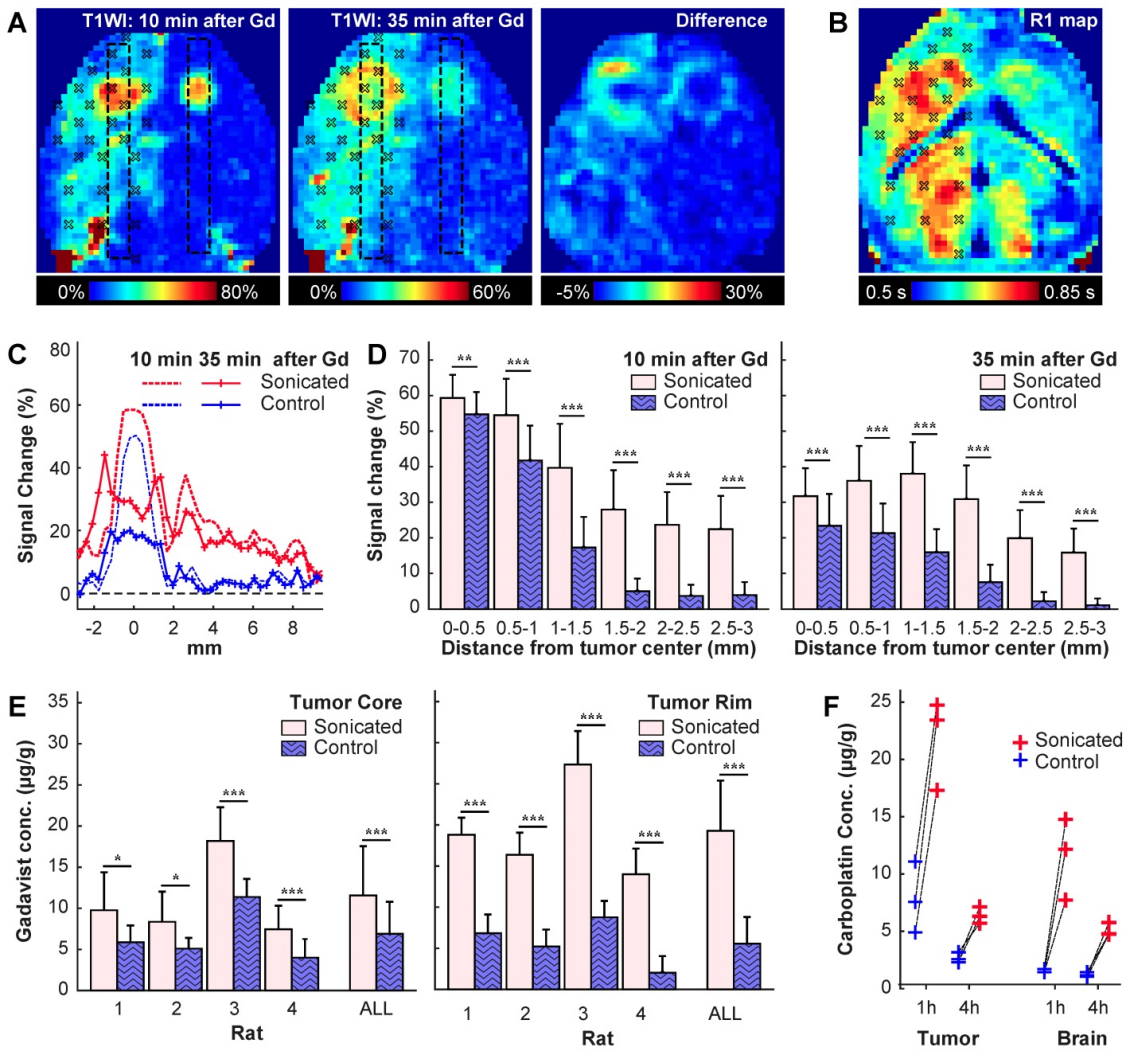
BBB/BTB disruption in a rat with bilaterally-implanted tumors. We sonicated 27 targets in one hemisphere. **(A)** Axial maps of signal changes in T1-weighted imaging (T1WI) at 10 and 35 min after injection of Gadavist, and the difference between these images. The sonication targets are noted. Note the apparent spread of the contrast from the tumor core to the outer margin. **(B)** Map of R1 relaxation in the same rat. **(C)** Plots showing mean signal enhancement in the regions indicated by the boxes in **(A)** at these times. The enhancement in the tumor center decreased substantially over time, but it increased in the surrounding margins. The enhancement in the normal brain evened out and remained at a similar level. **(D)** Plot of signal enhancement at different distances from the tumor center in four rats. **(E)** Gadavist concentrations estimated from the R1 maps at the core and in the surrounding rim. The estimated concentrations were higher in the tumor rim. **(F)** Carboplatin concentrations measured in the tumors and in an area of normal brain at one and four hours after the sonications. Drug concentration were measured post mortem via LC-MS/MS in biopsies each with a diameter of three mm. (* P<0.05; **P<0.01; ***P<0.001)

To study BBB/BTB disruption and corresponding carboplatin concentrations, we implanted two tumors (one in each side) in the striatum in six rats (**Figure 5**A). Three volumetric sonications (one in normal brain, two at different depths in the tumor) were applied to target 27 locations in the right hemisphere. The left hemisphere served as a control. At either one (N=3) or four (N=3) hours after sonication, the rats were euthanized and the brains extracted. Biopsies were obtained from the tumor and in the healthy brain, and blood samples (500 µl) were obtained. The concentration of intact carboplatin in the tissue and plasma samples were then measured by LC-MS/MS.

Evaluation of the patterns of signal enhancement in T1-weighted imaging allowed for visualization of the BBB/BTB disruption over time (**Figure 5**A, C-D). Immediately after injection of Gadavist, both tumors enhanced with a tiny central core that enhanced slightly more slowly (not shown). At 10 min after injection, the tumors appeared as small enhancing spots, and patchy enhancement was observed in the healthy brain. By 35 min, enhancement in the tumor center decreased markedly, while enhancement in a surrounding rim increased; enhancement in the healthy brain was more homogeneous at this time, and its magnitude decreased slightly.

The patterns observed in the rat shown in **Figure 5**A-C were evident when all rats were considered (**Figure 5**D-E). Signal enhancement was significantly higher in the sonicated tumors at 10 min and 35 min after sonication; at 35 min, signal enhancement at the tumor core decreased. Maps of R1 relaxivity (**Figure 5**B) were used to estimate Gadavist concentrations. Similar to the delayed enhancement in the T1-weighted images, higher concentrations, both absolute and relative to the control tumors, were found in the tumor margin (**Figure 5**E). In the tumor core, the estimated Gadavist concentration was 1.7 times that in the controls (11.6 ± 6.0 vs. 6.9 ± 3.9 µg/g); this ratio was 3.3 in the rim (19.3 ± 6.1 vs. 5.6 ± 3.2 µg/g). Additional images from this study are shown in **Figure S5**.

In agreement with the Gadavist findings, intact carboplatin concentrations were significantly higher in tumor and brain in the sonicated hemisphere than tissue resected from the opposing non-sonicated hemisphere (**Figure 5**F). At one hour after sonication, the ratio of the mean drug concentration (sonicated vs. non-sonicated) was 2.9 for tumor tissue [geometric mean (geometric %CV): 21.6 (20) vs. 7.4 (43) µg/g] and 7.3 for normal brain tissue [11.1 (34) vs. 1.5 (11) µg/g]. At four hours after sonication, the ratios were 2.4 for tumor tissue [6.4 (11) vs. 2.7 (16) µg/g] and 4.2 for normal brain [5.1 (11) vs. 1.2 (17) µg/g]. Relatively high drug levels persisted in tumor and normal brain tissue even though the concentration of intact carboplatin in plasma decreased more than 40-fold from 26.9 (23.9) µg/ml at one hour after dosing to 0.61 (19.6) µg/ml at four hours. The intact carboplatin concentrations are further summarized in **Table S3**.

### FUS-enhanced carboplatin delivery slowed tumor growth and prolonged survival in F98 glioma

**Figure 6:**
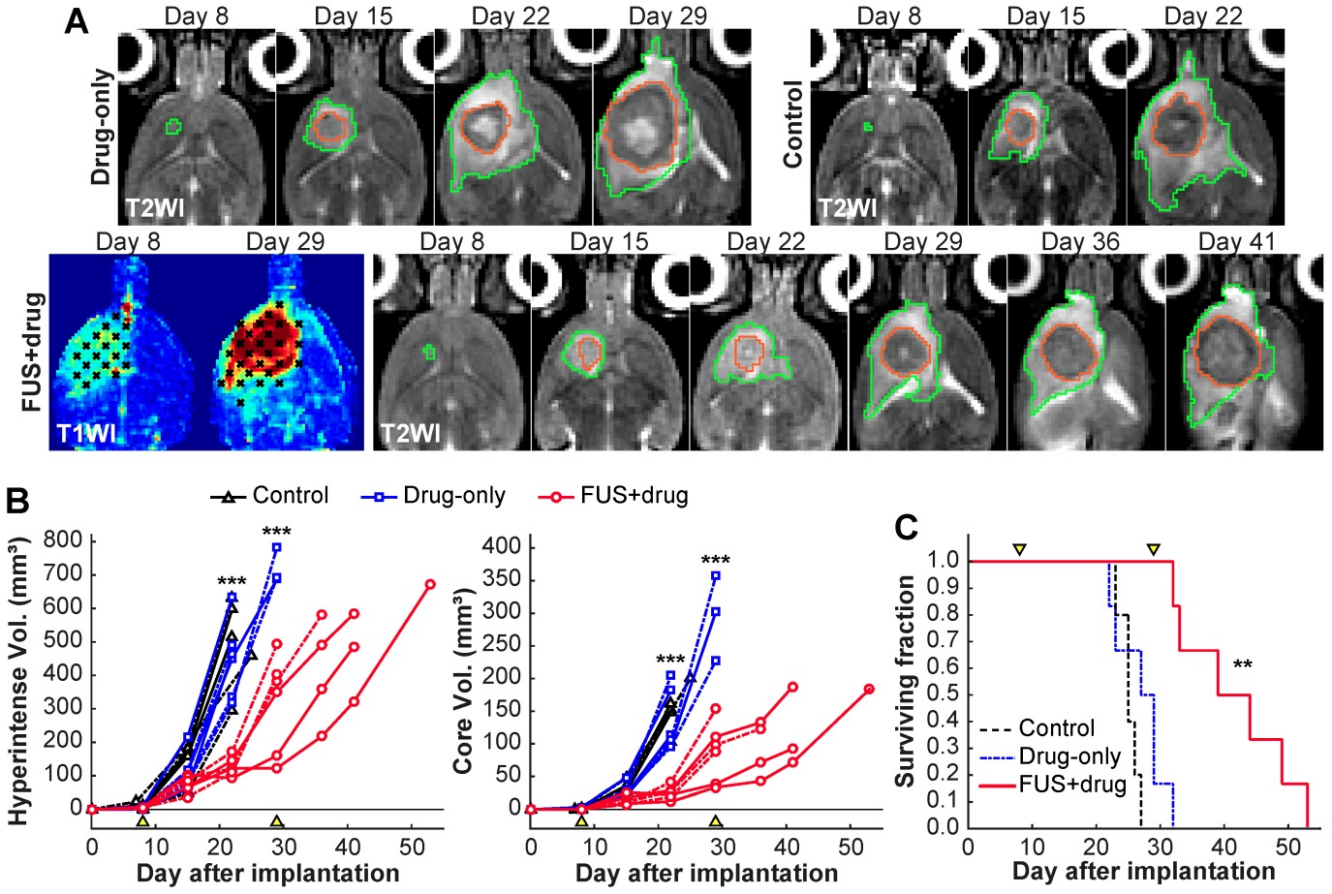
Efficacy study results. **(A)** Representative axial T2-weighted images (T2WI) of rats that received two treatments with carboplatin and BTB/BBB disruption on days 8 and 29, carboplatin alone on day 8, and no treatment. Maps of contrast enhancement in T1-weighted images (T1WI) are also shown for the FUS+drug rats. The segmentations of the hyperintense areas surrounding the tumor (green) and the tumor cores (orange) are shown. **(B-C)**. Tumor volumes and survival analysis. Arrowheads indicate treatment days. **(B)**. Volume of the hyperintense regions **(B)** and the hypointense cores **(C)** as measured in T2-weighted MRI. Tumor volumes of the FUS+drug rats was significantly (***p<0.001) less than the other groups at day 22 and the surviving drug-only rats at day 29. (Dashed lines: female rats) **(C)** Kaplan-Meier survival analysis. Median survival for the FUS+drug rats was significantly longer (**P<0.01) than the other groups.

We evaluated tumor growth and survival after enhanced carboplatin delivery. Rats either received BBB disruption and 50 mg/kg carboplatin (N=6), carboplatin alone (N=6), or no treatment (N=5). Representative contrast enhancement maps T2-weighted MRI obtained in this study are shown in **Figure 6**A. The region of contrast enhancement after Gadavist injection included the enhancing portion of the tumor in every treatment (**Figure S6**). One week after the first treatment, the tumors were similar in sizes. At two weeks and thereafter, the size of the tumors that received FUS and carboplatin was clearly smaller than the other experimental groups. The six FUS+drug rats all received a second treatment three weeks after the first. Three of six drug-only rats survived until this time.

The tumor core and surrounding hyperintense regions were segmented in multiplanar MRI. The volumes of these areas were significantly smaller in the FUS+drug rats on day 22 (P<0.001), the last day where all the animals were alive (**Figure 6**B). The tumor doubling time for the FUS+drug rats was significantly (P<0.05) increased compared to the other groups. The doubling time for the tumor core in the FUS+drug rats was increased by 94% and 126%, respectively, compared to the drug-only and control rats (Table 1). Histology was obtained in two rats from each experimental group. As is evident in the MRI, extensive tumor burden was observed histology upon histopathology evaluation (**Figure S7**). The tumor growth in the three males in the FUS+drug group was less than the three females, however the number of animals was insufficient to make statistical comparisons. A better response in the males is also evident in the weight of the animals (**Figure S8**). At the time of the second treatment, the male rats' weight was increasing, while the female rats had begun to lose weight.

Kaplan-Meier survival analyses are summarized in **Figure 6**C and Table 1. Survival was significantly longer in the FUS+drug rats than both the control rats (P=0.004) and the drug-only rats (P=0.006). The median survival in the FUS+drug rats was improved by 66% compared to the control rats and 48% compared to the drug-only rats. No significant difference was observed in survival between the control and drug-only rats (P=0.297).

## Discussion

These results demonstrate that controlled and repeated BBB disruption can be achieved in a rat model using a low-frequency clinical TcMRgFUS system. Further, the disruption was sufficient to enhance delivery of carboplatin to both the tumor and healthy brain and to prolong survival in the F98 glioma model.

We first evaluated the safety and repeatability of activelly-controlled BBB disruption in the normal brain. Disruption was achieved in every target in the sonicated volumes over 20 consecutive sessions, and closed-loop feedback control was successful in tailoring the acoustic power level to a pre-determined level of cavitation activity while minimizing wideband emission, a signature for inertial cavitation 23. This signature was detected in 8% of the sonicated targets with microbubbles. Previously, wideband emission was found to be correlated with vascular damage and petechiae 24, which were not found here. This finding may reflect the fact that the power was reduced immediately upon detection, limiting the potential damage to that produced by a single burst. Histology was obtained three weeks after FUS, and it is possible that any minor petechiae had resolved. The wideband emissions, as well as subharmonic emissions (reflecting microbubble activity near the inertial cavitation threshold), were most likely to occur at locations near the midline. We suspect that those targets included large blood vessels where microbubble concentrations were comparatively high, the bubbles were free to grow in size, and the vessel walls were thicker and perhaps more resistant to damage. More work would be necessary to confirm the impact of inertial cavitation in large and small vessels. Differences in vessel properties such as vascular density may also explain the observed variability in Gadavist delivery to different tissue structures, although other factors arising from absorption and reflection from the skull could have played a role.

We did not see evidence of significant tissue damage by either MRI or histology after three sessions of enhanced carboplatin delivery, in agreement with previous studies on BBB disruption with this device 6 and carboplatin delivery to the brain 25. The only damage we found was a scar in histology with dimensions less than 1 mm in one rat. We suspect that this result was due to vascular damage, or perhaps occlusion or spasm, resulting in a tiny ischemic area. Importantly, we did not observe MRI-evident delayed tissue damage or brain tissue loss that has been observed in studies with repeated BBB disruption that employed a large microbubble dose 26 or that used uncontrolled sonication settings 27. While we only examined long-term effects in histology and thus could not observe inflammation or other reversible changes, a lack of edema in T2-weighted imaging at 48-72 hours suggests any inflamatory response was likely minimal. Overall, these findings are promising for the prospect of safely using FUS to deliver carboplatin to areas surrounding a macroscopic tumor in the intact brain that contain infiltrating microscopic tumor cells in order to decrease the risk of recurrence.

Examination of BTB disruption revealed delayed enhancement and higher Gadavist concentrations in the tumor compared to the core. We suspect that this pattern was the result of high interstitial tumor pressure, which produces a radial outward convection of extravasated drug 28. In the normal brain, we observed diffusion away from the focal targets between 10 and 35 min, leading to a homogenous enhancement pattern. It would be interesting to monitor this diffusion over a longer time to better optimize the spacing needed to achieve a uniform drug concentration over a large volume. Relatively high levels of intact carboplatin were sustained in tumor and normal brain tissue for at least four hours after BBB disruption. In fact, at four hours after dosing, the concentrations of intact carboplatin in tumor and normal brain tissue exceeded the corresponding concentration in plasma by factors of 10.5 and 8.4, respectively. In contrast, carboplatin was largely cleared at four hours in the control tumors, and little drug was detected in the normal non-sonicated brain. We measured similar levels of carboplatin in the brain and the tumor at four hours, a finding that is consistent with previous measurements in this tumor model at 2.5 hours after intravenous carboplatin 29. The ratio of carboplatin in the brain (sonicated vs. control hemispheres) measured here (7.3 times) is also similar to that obtained in a monkey (5.2 times) an hour after BBB disruption with an implanted ultrasound device 30 and the 4.2-fold increase found in mice after disruption with 1.05 MHz ultrasound 12.

Finally, we evaluated the efficacy of this enhanced carboplatin delivery to a rat glioma model. Our findings are consistent with those by Drean et al., who examined weekly treatments with carboplatin and FUS-mediated BBB disruption in two orthotopically xenografted human glioma cell lines in mice 12. They observed an increase in median survival time (IST) of 46-63% compared to untreated controls, similar to the 66% found in the present study. Along with our results, that study provides additional support for the clinical evaluation of FUS-enhanced carboplatin delivery to brain tumors.

Here we used F98 tumors, an infiltrative cell line that is highly aggressive and minimally immunogenic 31. While differences in cell numbers, implantation sites, treatment schedules and other factors make exact comparisons to other studies challenging, our results appear to be favorable in comparison to previous work in this tumor. Timbie et al. examined delivery of cisplatin nanoparticles via FUS-mediated BBB disruption and found an increase in median survival time (IST) of 15% 32. Charest et al. tested intra-arterial delivery of carboplatin (20 mg/kg) in conjunction with osmotic BBB disruption with mannitol 33. They found an IST of 38% over controls, which was not significant. Côté et al. found a significantly-improved IST of 46% after BBB disruption using vaso-active agents 34. The IST of 66% found here is comparable to previous studies using convection-enhanced delivery (CED) of carboplatin, where reported IST ranges from 58-64% 35-37. However, those studies had some long-term survivers, which we did not. CED is advantageous since it can achieve very high local drug concentration and avoids systemic side effects. However, it is invasive, and it can be challenging to achieve effective delivery to large volumes. Substantially longer survival times have been achieved in this rat model by combining CED of carboplatin with ionizing radiation 33,35-37. It would be interesting to combine radiation with FUS and BBB disruption to see if similar synergistic effects occur. A few studies have reported longer survival in F98 tumors with investigational drugs, cell therapies, or immune therapies 38-41. Such treatments could potentially be improved with FUS-induced enhanced delivery as well.

With a doubling time of only 3-4 days without treatment, however, the F98 model may not have been optimal for the treatment schedule used in this study. We suspect that the clinically-used protocol of three weeks between treatments may have resulted in too extensive a tumor burden for enhanced carboplatin delivery to have a meaningful effect after the second treatment. While a small effect was perhaps evident in some rats, future work should use a slower-growing tumor or perhaps a different treatment schedule to confirm that the treatment effects of FUS-mediated carboplatin delivery continues over multiple treatments. The rapid growth of the F98 model also prevented detailed analysis of treatment effects on infiltrating tumor cells. It would be interesting to examine the tumors in the different groups in histology at earlier times after treatment to see if enhanced carboplatin delivery reduces infiltration into brain regions with an intact BBB.

This study had other limitations. First, the number of animals was relatively small. While the main results were clear and statistically significant, rare events and other factors could have been missed. For example, there appeared to be signs of different responses between the male and female animals in the efficacy study. We suspect that this may have been due to differences in their weight (i.e. it may be necessary to use a different scaling factor to estimate human equivalent doses) or perhaps differences in renal clearance. Another limitation was potential errors in our R1 maps due to Gadavist diffusion over the long scan time. The acoustic emissions controller settings could also be better optimized to avoid overshoot. While we demonstrated that the contoller maintains a safe exposure, additional demonstration of the ability of the feedback controller considering the thickness and variabiltiy of the human skull also should be shown. Such validation is challenging in rats, where inter-subject variability is low. Finally, we only examined drug concentrations at one stage of tumor growth; previous work has shown a dependence on permeability and drug concentrations on this factor 42.

It is important to perform preclinical studies with the same device that will be used clinically. One can use larger animal models 5,7,8, but such experiments are expensive and time-consuming. Further, disease models in large animals are limited. Regulatory agencies may not accept preclinical data obtained with different equipment, particularly when they are performed at a different FUS frequency. These results are therefore promising for future work to evaluate different chemotherapies or other treatments for brain tumors and other disorders that have rat models.

## Conclusions

This work shows that ExAblate Neuro low-frequency clinical TcMRgFUS system can safely be used to repeatedly and reliably disrupt the BBB in an aggressive rat model. An integrated closed-loop control system based on recordings of the increase in acoustic emissions produced by the presence of Definity microbubbles ensured that BBB disruption occurred without significant vascular damage. The delivery of carboplatin to the brain was not neurotoxic and was sufficient to significantly prolong survival compared to treatment with the drug alone in the F98 rat glioma model.

## Methods

### Animals

The experiments were performed using either Sprague-Dawley (4 male, 3 female; safety study) or Fischer CDF rats (11 male, 12 female; tumor studies). They were anesthetized with isoflurane (typically 2-3%) and air. The fur on the top of the head was removed with clippers and depilatory cream, and the tail vein catheterized. For the sonications, the animals were placed in an acrylic stereotactic frame that was constructed in-house and placed supine on the TcMRgFUS system. All experiments were approved by the Institutional Animal Care and Use Committee at Brigham and Women's Hospital.

### Equipment

The ExAblate Neuro low-frequency TcMRgFUS system (InSightec) was used for the experiments. This system consists of a 1024-element hemispherical transducer (diameter: 30 cm) that operates at 230 kHz, an ultrasound driving system, a cavitation monitoring system, and a water cooling/degassing/circulating system 10,11. For these experiments, the transducer was placed on its side so that it could be filled with water like a bowl (**Figure 1**A). The phased array transducer was used to electronically steer the focal point to different targets. No aberration correction was used to compensate for the rat skull. The center axial plane of the brain was positioned at a depth approximately one cm above the geometric focal plane of the TcMRgFUS transducer.

The TcMRgFUS system was integrated into a 3T clinical MRI machine (Signa HDxt, GE Healthcare). A rectangular receive-only surface coil (dimensions: 5×6 cm; constructed in-house) was used during the experiments. The coil was mounted on a plate that was partially submerged below the water level of the TcMRgFUS device (**Figure 1**A). The rat was placed in a stereotactic frame that was mounted on top of this plate so that the only the top of the head was in water. In this setup, the brain was in the center of the coil while the body was kept dry. For pre- and post-FUS imaging, the water was drained from the transducer, as the loading and/or coupling of the water bath with the MRI coil reduced the SNR significantly. Body temperature was maintained with a heated water blanket.

MRI was used to plan the treatment, evaluate the BBB disruption, monitor tumor progression, and detect tissue damage. Before sonication, T2-weighted imaging was acquired to visualize the tumor and any tissue damage evident from a previous session, and T2*-weighted imaging was acquired to plan the treatment. After sonication, T2*-weighted imaging was collected. Next, T1-weighted imaging was acquired before and after administration of MRI contrast (Gadavist, Bayer HealthCare Pharmaceuticals, 0.125 mmol/kg). Maps of R1 relaxation were then acquired, followed by additional T1-weighted imaging acquisition to evaluate delayed contrast enhancement. In the safety study, T2-weighted imaging was acquired at 48 or 72 h after sonication to detect tissue damage. In the efficacy study T2-weighted imaging was acquired weekly to monitor tumor progression.

### MRI acquisition

Before sonication, an axial 3D T2*-weighted spoiled gradient echo sequence (TR/TE: 33.3/19.0; flip angle: 15°; FOV: 8×8×2.1 cm; matrix: 256×256×30; bandwidth: ±15.6 kHz; averages: 1) was used to select the sonication targets. This imaging was repeated immediately after the last sonication to detect petechiae. After sonication, an axial T1-weighted fast spin echo (FSE) sequence was obtained (TR/TE: 500/13.8 ms; ETL: 4; FOV: 8×8 cm; slice thickness: 1.5 mm; matrix: 256×256 bandwidth: ±15.6 kHz; averages: 4). This imaging was repeated five times (four axial, one sagittal acquisition) after IV injection of 0.125 mmol/kg Gadavist (Bayer HealthCare Pharmaceuticals, Inc.). Finally, a FSE sequence repeated with multiple TR's was obtained to create maps of the R1 relaxation rate (TR: 6000/3200/1600/800/400/200/100 ms; TE: 13.4 ms; ETL: 3; FOV: 9×9 cm; slice thickness: 2 mm; matrix: 256×256; bandwidth: 15.6 kHz; averages: one for TR=6000/3200/1600 ms, 2 for TR=800/400 ms, 4 for TR=200/100 ms). We obtained another acquisition with the T1-weighted FSE sequence after R1 mapping to observe Gadavist clearance and potential delayed enhancement.

T2-weighted FSE images (TR/TE: 4000/81.7 ms; ETL: 12; FOV: 9×9 cm; slice thickness: 2 mm; matrix: 256×256 bandwidth: ±15.6 kHz; averages: 1) were obtained weekly to measure tumor growth and used to confirm targeting before sonication in the tumor experiments. This imaging was also acquired at 48-72 hours after FUS in the safety study and before the sonications in the second and third sessions in to evaluate whether any edema was present.

### MRI processing

Maps of the R1 relaxation rate were created using the method outlined elsewhere 43. The “nlinfit” command in Matlab was used for the nonlinear regression. Three axial images were obtained. Different tissue structures were manually segmented in the center slice by one user (NM). Maps of differences in R1 between the right and left hemispheres were created from these segmentations. To conclude that BBB disruption occurred, we compared regions in these ΔR1 maps that were within ±1.5 mm of the individual targets, along with corresponding targets in the contralateral hemisphere. This comparison was evaluated both for individual tissue structures and for the entire region included in the volumetric sonications. The R1 relaxation rate, along with the relaxivity of Gadavist - 4.44 s^-1^·mmol^-1^ 22 - was used to estimate differences in tissue concentrations of the contrast agent between sonicated and non-sonicated areas. For experiments in tumors, we estimated absolute concentrations using R1 measurements obtained in one animal before administration of Gadavist.

Contrast enhancement in the T1-weighted images was calculated as a percent increase relative to imaging obtained before Gadavist injection. It was estimated in five planes, and a maximum intensity projection was performed after segmenting the brain. The mean signal enhancement in these projections was measured for the areas covered by the volumetric sonications and corresponding locations in the contralateral hemisphere.

Tumor volume was assessed in multiplanar T2-weighted imaging by one author (NM), who segmented the tumor core, which was typically hypointense as well as the surrounding hyperintense areas. The tumor volume as a function of time was fit to an exponential to estimate the doubling time. This fit used the “nlinfit” command in Matlab.

### Sonications

In each rat, either 18 or 27 overlapping targets were sonicated with an aim of disrupting the BBB in a volume that covered most of the cerebrum (safety study) or that covered the tumor and a surrounding margin in the right hemisphere at a central axial plane. These targets were selected with a 2 mm center-to-center spacing and arranged to conform to the shape of the rat brain (**Figure 1**B). Before the sonications in each rat, the pre-FUS T2*-weighted MRI were manually registered to a template using software developed in-house. Each volumetric sonication targeted 9 locations (**Figure 1**B); 2 or 3 sonications were delivered to each rat. The sonications were first delivered without microbubbles to establish a baseline and to confirm a lack of cavitation activity (total duration: 30 s), and then were repeated with microbubbles (total duration: 75 s). Definity microbubbles (Lantheus Medical Imaging) were administered at the start of the sonication as a bolus injection at the approved clinical dose for ultrasound imaging, 10 µl/kg (~1.2×10^8^ microbubbles/kg). To facilitate the injections, the agent was diluted 10:1 in PBS and was followed by a 200 µl injection of saline. The volumetric sonications consisted of 5 ms bursts applied sequentially to the nine targets at an interval of 101.6 ms; the pulse repetition frequency for each target was thus 1.1 Hz. A delay of at least two minutes ensured that most of the microbubbles had cleared at the start of each sonication.

### Feedback control

The acoustic power level was controlled in real-time based on analysis of acoustic emissions 44-46. Sonication began at an acoustic power level of 0.16 W and was not allowed to exceed 0.39 W. Based on calibrations performed with a 4 mm diameter calibrated omni-directional needle hydrophone (model TC4038, Reson), this power range corresponds to estimated peak pressure amplitudes of 119-186 kPa in water. Emissions were recorded with two hydrophones. One was integrated into the TcMRgFUS device and was resonant at the subharmonic of the fundamental FUS frequency (115 kHz). The second hydrophone, placed at the bottom of the TcMRgFUS transducer, was elliptical (5×3 cm), air-backed, and had a resonant frequency of 660 kHz. The power for each target in the volumetric sonication was independently modulated during the exposures based on the strength of the harmonic emissions at 460 and 690 kHz. If either wideband or subharmonic emissions were detected, the power level was reduced by 25% and fixed for the remainder of the sonication 44.

### Acoustic emissions acquisition

The range of power levels used, and the thresholds and parameters used for the controller were determined in pilot studies (data not shown) and aimed to be relatively conservative while minimizing false detection. Before each sonication, spectra were obtained for 3.5 s to determine the noise floor. The controller began 8 s after the start of the sonication to allow the microbubbles time to reach the brain. It used a proportional controller based on the mean signal strength at the second and third harmonics to modulate the power until a pre-determined level of harmonics (between 6-7.7 dB above the noise floor) was achieved. If the harmonic increase (*H*) for a burst was outside of this range, the power was changed based on the following equation:


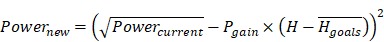


where the constant *P_gain_* was 0.0167. This controller assumed that the strength of the harmonic enhancement in dB was proportional to the pressure amplitude 45.

Since the microbubble concentration decreases after injection, the controller was programmed to never increase the power after 25 s. At this time, it set the power to the mean value of all bursts where *H_goal_* was achieved. If at any time subharmonic emission was detected by the first hydrophone or broadband emission by the second, the power level was reduced by 25%, and the power level was fixed for the remainder of the sonication 44.

Subharmonic emission at 115 kHz and harmonic emissions at 460 and 690 kHz were calculated in 10 kHz bins. Broadband emissions were calculated in a 40 kHz bin centered at 0.66 kHz, the resonant frequency of the second hydrophone. During the first 8 s of each sonication (before the microbubbles arrived in the brain), a second noise floor measurement was obtained during bursts applied at 0.16 W. The threshold for subharmonic emissions was defined to be 3.2 standard deviations above this noise floor; it was set to 3.9 standard deviations above this noise floor for broadband emissions.

### Carboplatin administration

Carboplatin (Teva Pharmaceuticals) was injected intravenously at 50 mg/kg over a period of approximately 90 s. This dose corresponded to a human equivalent dose of 300 mg/m² (using a conversion factor of six); it and the treatment frequency (every three weeks) were selected to be within the range used clinically 47,48 and that result in limited toxicity in rats 49. In the safety study, the drug was administered between the two volumetric sonications to investigate whether the order of BBB disruption and drug was important in case any tissue damage was evident. The order of the sonications was reversed in half the animals. In the tumor studies, the drug was administered immediately after the last sonication.

### Safety study

In six rats, we applied two volumetric sonications at one depth in the brain. An additional rat was sonicated in three locations in one hemisphere; in this rat we sonicated at two depths in the striatum. At 48 or 72 h after sonication, T2-weighted images were obtained to evaluate whether the sonications produced edema. Each rat received three sessions of FUS and carboplatin spaced three weeks apart. The animals' weight was recorded regularly, and any adverse effects on the animals' appearance or behavior were noted.

Three weeks after the last session, the animals were deeply anesthetized and euthanized via transcardial perfusion with formalin. This time was selected to detect potential delayed MRI-evident damage that has been observed by others with a high microbubble concentration 50. The brain was then removed and immersed in formalin for 24-48 h. It was cut into three axial blocks and photographed. Using these pictures and the MRI as a guide, selected blocks were paraffinized, cut in 5 µm sections, and stained with H&E. One rat died before the sonications in the third session due to overfilling the transducer with water (operator error). This rat was not included in the histology examination. H&E stained sections were scanned at 10× resolution. They were evaluated by one author (NV) without knowledge of experimental group or which hemisphere was sonicated.

### Tumor studies

Wild-type F98 cells (passage number six, provided by Rolf F. Barth 31 at the Department of Pathology, The Ohio State University, Columbus, OH) were cultured in Dulbecco's modified Eagle medium (1×) supplemented with 10% FBS and 0.1% Penicillin Streptomycin in a humidified incubator with 5% CO2 at 37°C. Following the surgical procedure as previously described 45, a 4-μL cell suspension (2 × 10^4^ cells) was injected into the caudate putamen 3.5 mm from the dura surface using a 10-μL gastight syringe (Hamilton) in Fischer rats. Animal behavior was monitored daily after surgery and the sutures were removed 5 days later.

To compare delivery of carboplatin to tumor and healthy brain, we implanted two tumors (one in each hemisphere) in six rats. Eleven days after implantation, we disrupted the BTB/BBB in one hemisphere. Two volumetric sonications at two depths covered the tumor and a surrounding margin (volumes T1-2 in **Figure 1**B); a third sonication was applied at a single depth in a region of healthy brain (volume S2 in **Figure 1**B). Rats were euthanized either 1h (N=3) or 4h (N=3) after sonication. The brains were extracted and biopsies (diameter: three mm; approximately 50 mg) were obtained from the tumor and normal brain. The samples were blotted with filter paper, rinsed in ice cold PBS and frozen at -80°C. Blood samples (500 µL) were acquired, heparin was added, and centrifuged. Plasma was extracted and frozen at -80°C. Carboplatin concentrations were then measured as described below.

In the efficacy study, a tumor was implanted in the right hemisphere, and the rats were divided randomly into three groups (control, N=5; drug-only, N=6; FUS+drug, N=6). On day 8 after tumor implantation all animals were anesthetized and imaged to confirm tumor growth. The FUS+drug rats received FUS and microbubbles to disrupt the BBB as described above followed by carboplatin administration. Drug-only rats received carboplatin at this time. A second treatment was administered three weeks later. We did not include a FUS-only group since multiple studies have found no survival improvement with BTB/BBB disruption alone 2,4,51. The animals were imaged weekly to monitor tumor growth. Animals were euthanized when they exhibited severely impaired activity, weight loss exceeding 20% within one week, or tumor core dimensions exceeding 10-11 mm. The brains were removed and prepared for histology as described in the safety study. The animals' weight was recorded regularly, and any adverse effects on the animals' appearance or behavior were noted. The sonications were administered at two depths to cover the tumor and a surrounding rim (volumes T1-T2 **Figure 1**B for first treatment; T1-T3 in the second).

### Determination of Intact Carboplatin

Intact carboplatin was measured in plasma by high performance liquid chromatography with tandem mass spectrometric detection as previously reported with minor modifications 52,53. Frozen tissue samples were thawed, rinsed three times with ice-cold phosphate buffered saline, gently blotting with filter paper between each rinsing, and weighed in a microcentrifuge tube. After adding ice-cold water at a volume equivalent to 6-times the tissue weight, assuming a density of 1.0 g/cm³, the tissue was homogenized for 4 min using an Ultra-Turrax T8 disperser with an S8N-5G dispersing element (IKA Works, Inc., Wilmington, NC). The homogenate was sonicated for 5 min, subjected to three freeze-thaw cycles, and centrifuged (12,000 g, 10 min). Tissue homogenates were assayed in the same manner as plasma samples. The lower limit of quantitation was 1.0 ng/mL for determination of the drug in plasma and 7.0 ng/g for tumor tissue.

### Data analysis

All analysis was performed in Matlab (Mathworks). Statistical comparisons on MRI and acoustic emissions findings were made using unpaired t-tests; P<0.05 was considered significant. Carboplatin concentrations are reported as the geometric mean (geometric %CV), and comparisons between sonicated and control tissue samples used a paired two-tailed t-test of log transformed data. Survival times estimates and median survivals were determined using the method of Kaplan and Meier. A log-rank test was used to calculate P values derived from statistical analysis of Kaplan-Meier survival curves.

## Supplementary Material

Supplementary figures and tables.

## Figures and Tables

**Table 1:** Tumor volumes and survival analysis

		Doubling time (days)		Survival (days)			Median	Hazard	
Group	N	HIV	Core	Range	Median	Mean	IST	Ratio	P
Control	5	3.9 ± 1.3	3.1 ± 0.4	23-27	25	25.2 ± 1.5	-		-
Drug-only	6	4.3 ± 1.5	3.6 ± 0.9	22-32	28	27.0 ± 3.8	12.0%¹	1.3¹	0.297¹
FUS+drug	6	7.5 ± 2.7^*^	7.0 ± 2.8^*^	32-53	41.5	41.7 ± 8.5	66.0%¹, 48.2%²	1.9¹, 1.5²	0.004¹, 0.006²

^*^ P<0.05 compared to both control and drug-only groups ¹ Compared to control group ² Compared to drug-only group HIV: Hyperintense volume; IST: Increase in median survival time

## Abbreviations

BBB: blood-brain barrier
BTB: blood-tumor barrier
CED: convection-enhanced delivery
FUS: focused ultrasound
MRI: magnetic resonance imaging
TcMRgFUS: transcranial MRI-guided focused ultrasound
