# Supplementary material for: Acoustic feedback enables safe and reliable carboplatin delivery across the blood-brain barrier with a clinical focused ultrasound system and improves survival in a rat glioma model
Source: Theranostics. 2019 Aug 14;9(21):6284–99. doi: 10.7150/thno.35892 (PMC6735504; doi:10.7150/thno.35892)
Supplement: Supplementary file 1 — Supplementary figures and tables. [file thnov09p6284s1.pdf]

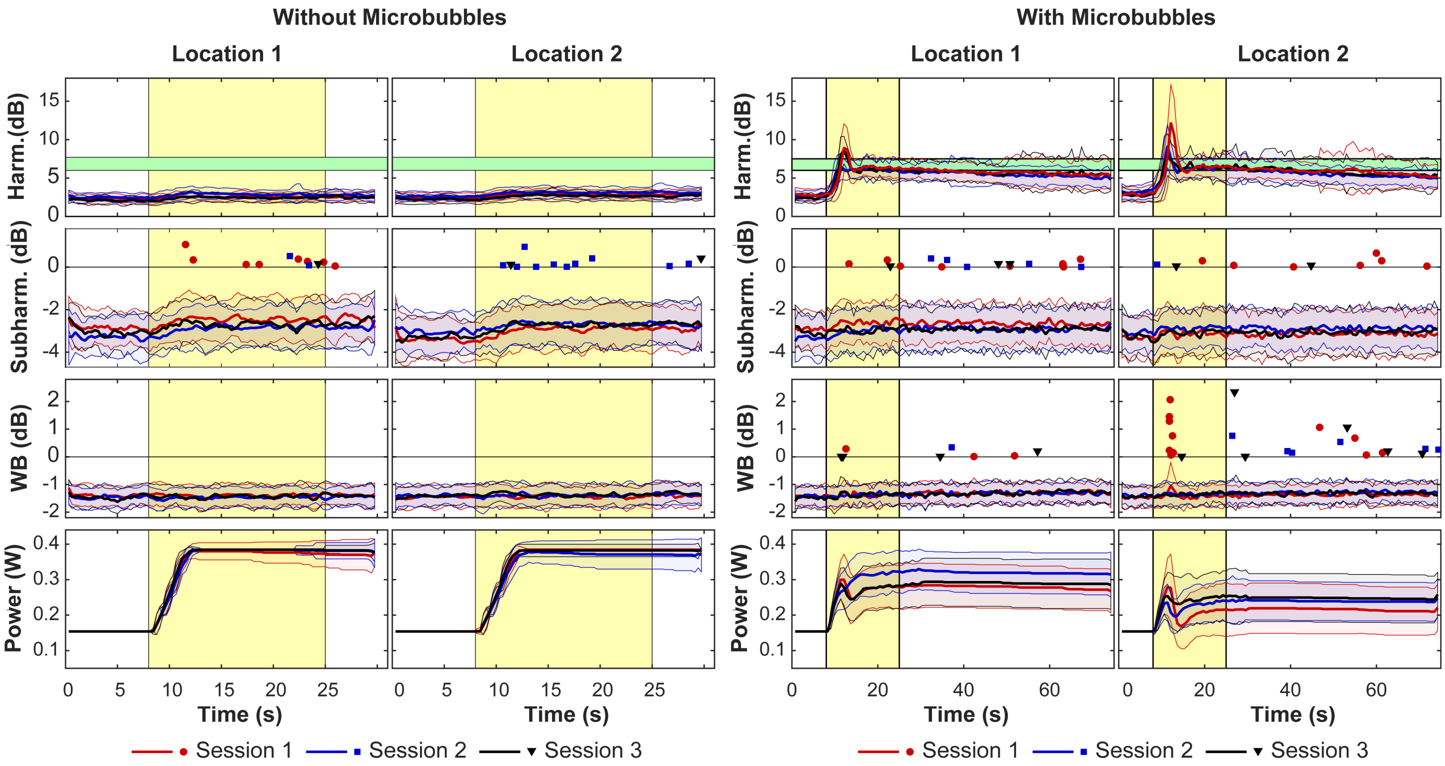

Figure S1: Mean acoustic emissions and acoustic power vs. time for all animals in the safety study. Harmonic emissions were the basis to control the power level at each target to achieve a pre-determined level between 6-7.7 dB above the noise floor. The controller began 8 seconds after the start of the sonication to allow time for the microbubbles to reach the brain; it was not allowed to raise the power after 25 seconds to avoid overexposure as the microbubbles were cleared from circulation. With microbubbles, the levels achieved were similar for the two locations targeted, for the three sessions, and for the different animals. Overshoot was evident, particularly in locations 2. Wideband (WB) and subharmonic emissions, plotted here in dB relative to the thresholds used to trigger a reduction in acoustic power, rarely occurred. The triggers that occurred are noted by the filled symbols. These subharmonic emissions occurred during sonication with and without microbubbles and had magnitudes close to the threshold. Supra-threshold wideband emissions did not occur without microbubbles; with microbubbles the strength of these emissions were variable. The acoustic power varied substantially from sonication to sonication and among the different sessions. However, its average trajectory over time was similar for the different sessions. Location 2 consistently required a lower power level. (Thick lines: mean values; thin lines/shaded areas: ± one standard deviation).

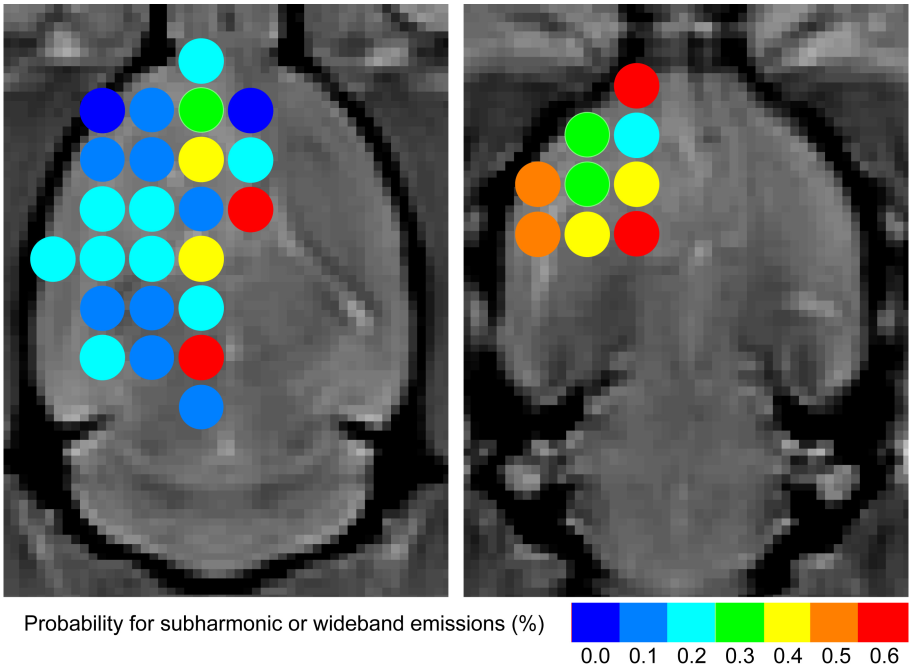

Figure S2: Axial MRI superimposed with the locations of the targets used in the study. At each target, the percent of bursts applied where subharmonic or wideband emissions were detected are shown. While this value never exceeded 1% at any target, it appeared that the probability was greater in the locations adjacent to the midline and at the targets administered at a deeper plane (right subplot) that were used in the tumor studies. Emission recordings from both the safety and tumor studies were used to generate this figure.

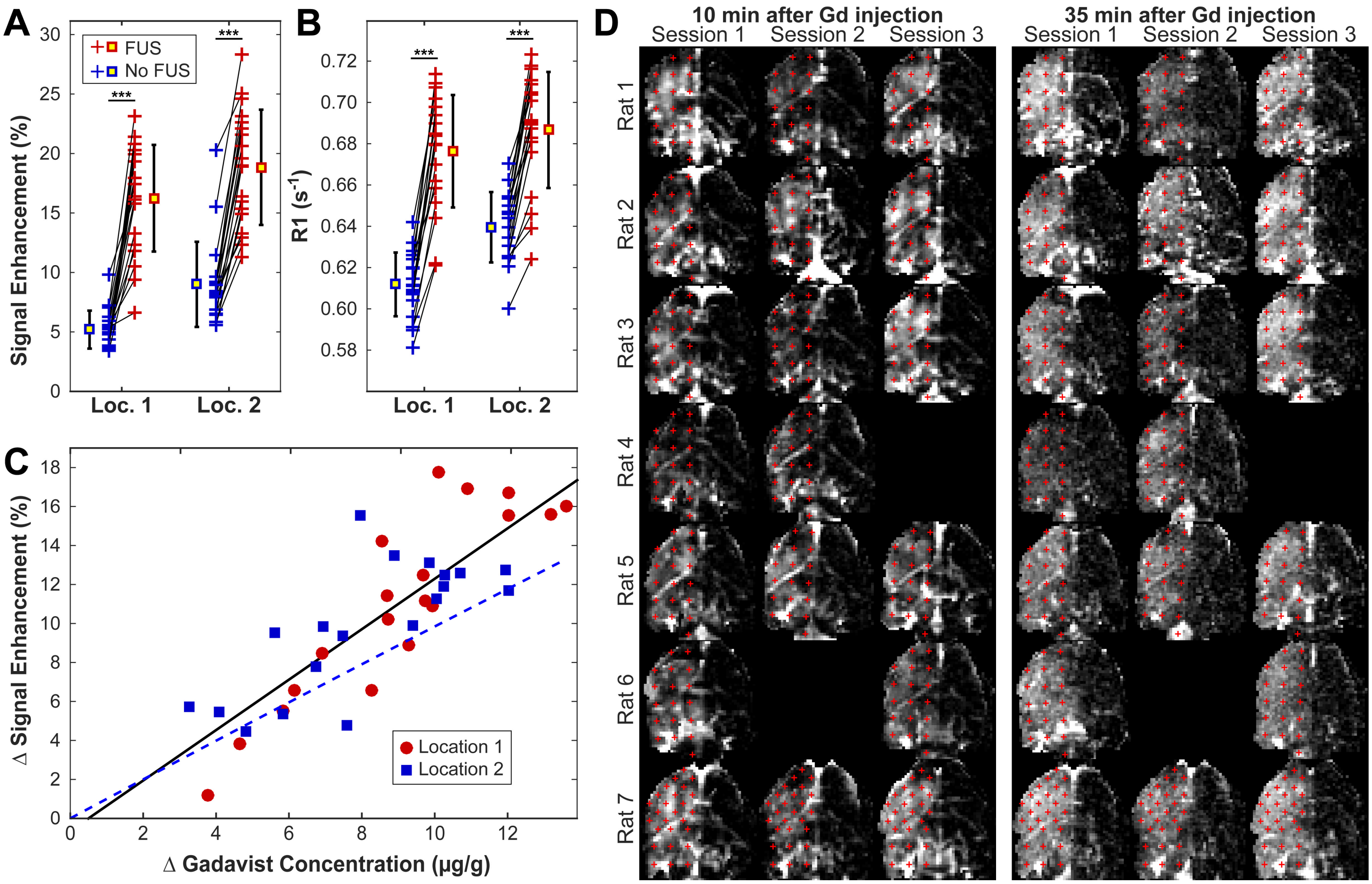

Figure S3: Delivery of MRI contrast agent in the safety study after volumetric sonication at two locations in one hemisphere. **(A)** Signal enhancement in T1-weighted imaging relative to pre-contrast imaging. **(B)** Estimates of R1 relaxation. **(C)** Correlation between changes in signal enhancement in T1-weighted imaging and R1 relaxation estimated via R1 calculations. A good correlation was observed (R^2: 0.69); linear regression plotted as a solid line. The dotted line shows the expected signal intensity change in this range of Gadavist concentrations, based on the equation for the signal intensity of a spin-echo sequence, the sequence parameters, and the R1 and R2 values of brain tissue. **(D)** Maps of signal enhancement for every rat in the safety study at two different times after injection of Gadavist (Gd). At 10 min after injection, the enhancement was spotty, and enhancement at individual targets are evident. At 35 min after injection, the enhancement was more spatially uniform. *(*** P<0.001)*

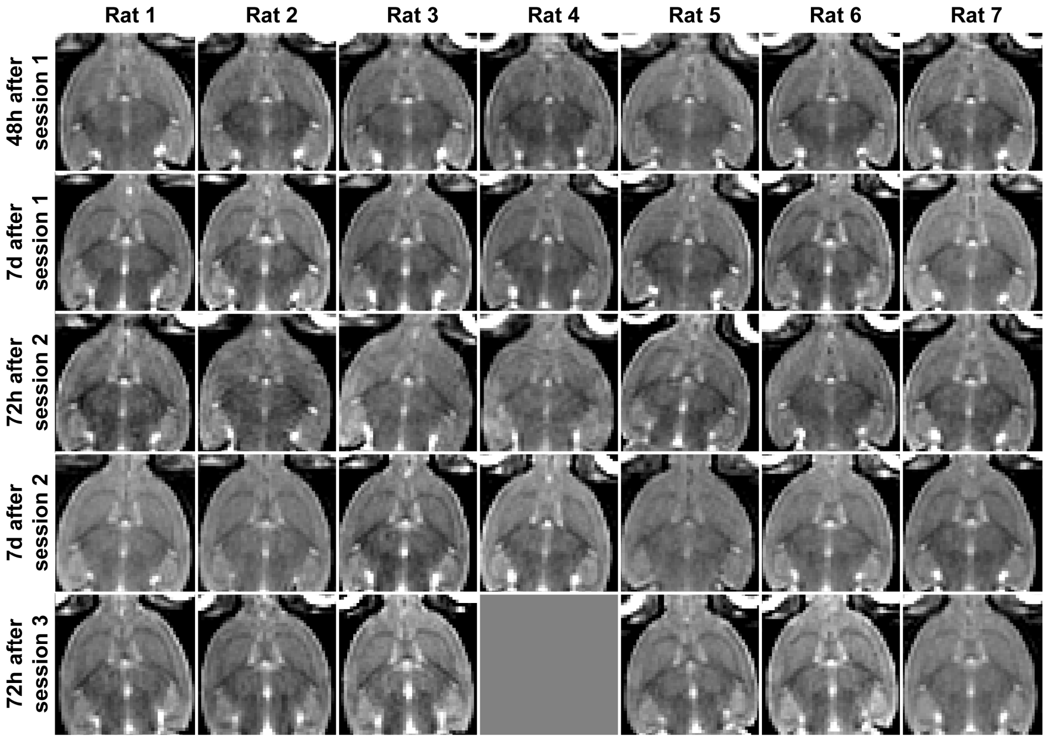

Figure S4: Axial T2-weighted MRI from the safety study obtained 48 or 72h and weekly after each session. No abnormalities were found.

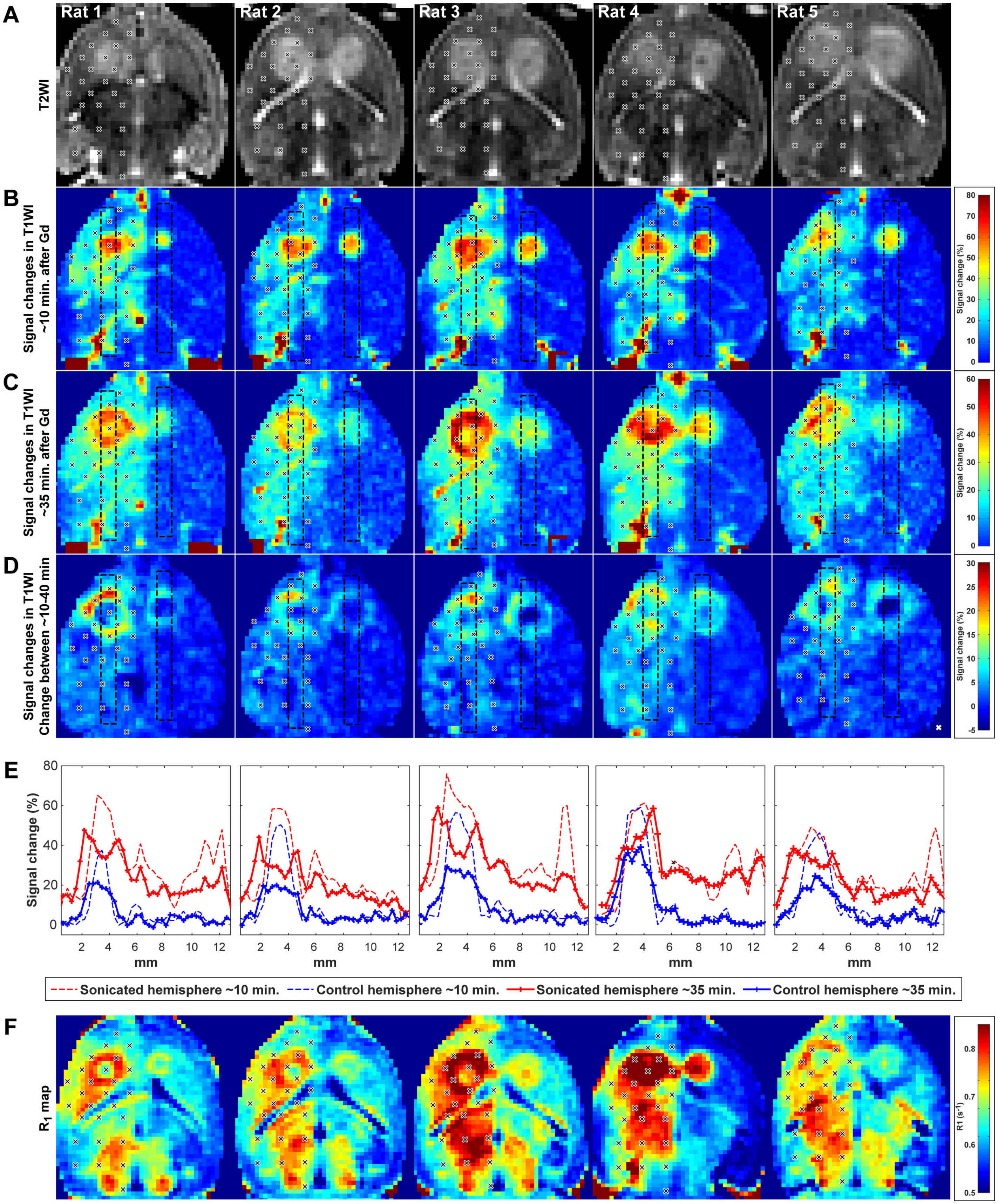

Figure S5: MRI obtained in rats with bilaterally-implanted tumors. **(A)** Pretreatment T2-weighted (T2WI) showing the tumor and the 27 sonication targets in one hemisphere. **(B-C)** Maps of signal enhancement in T1-weighted imaging (T1WI) at approximately 10 and 35 min after Gadavist injection. **(D)** Signal changes between these times showing delayed enhancement at the tumor margins. **(E)** Mean signal enhancement at these two times in the regions indicated in **(B-D)**. **(F)** Maps of R1 relaxation obtained in these rats. Note that Rat 4 received a larger Gadavist dose (operator error), and measurements obtained from it were not used in **Figure 5**C. Imaging data from another animal in this study was excluded due to artifacts.

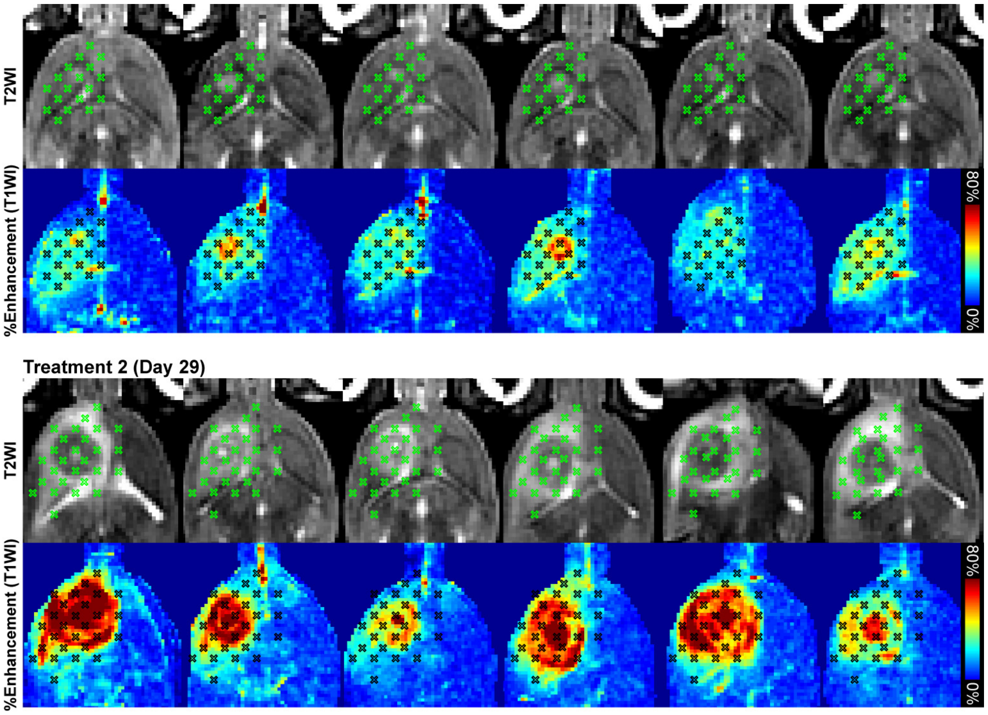

Figure S6: Axial T2-weighted imaging (T2WI) and maps of Gadavist-induced signal enhancement in T1-weighted imaging (T1WI) obtained before and after two treatments with BTB/BBB disruption and carboplatin. The locations of the individual sonication targets are indicated. Note that targets were staggered over two depths in the brain. FUS was applied with two (treatment 1) or three (treatment 2) volumetric sonications each consisting of nine individual targets. The three left-most rats were males.

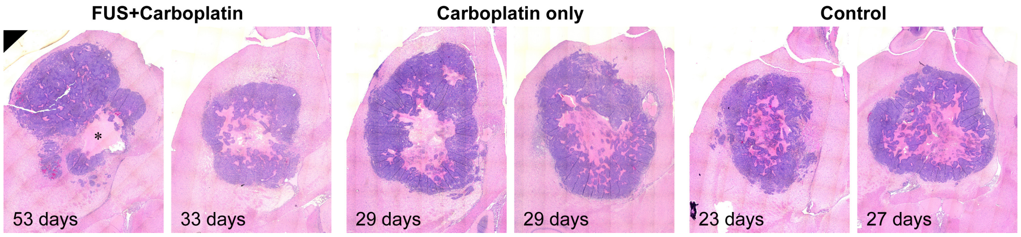

Figure S7: Scans of H&E stained sections of F98 from the three experimental groups. In each animal, a necrotic region at the center of the tumor is evident that was surrounded by densely-packed tumor cells. Perivascular clusters of tumor cells surrounded capillaries in the central necrotic area. In the drug-only and control rats, extensive invasions into contiguous normal brain were evident with islands of tumor cells at varying distances from the main tumor mass. In the rat that received BBB disruption and who survived 53 days, most of the tumor growth appeared to be from an area on the edge of the site of the primary tumor site, perhaps suggesting under-treatment in that area. In the rat that survived 33 days, the central necrotic region was surrounded less densely-packed tumor cells. One part of the invading edge of the tumor had no tumor cells at all, suggesting necrosis (arrow)

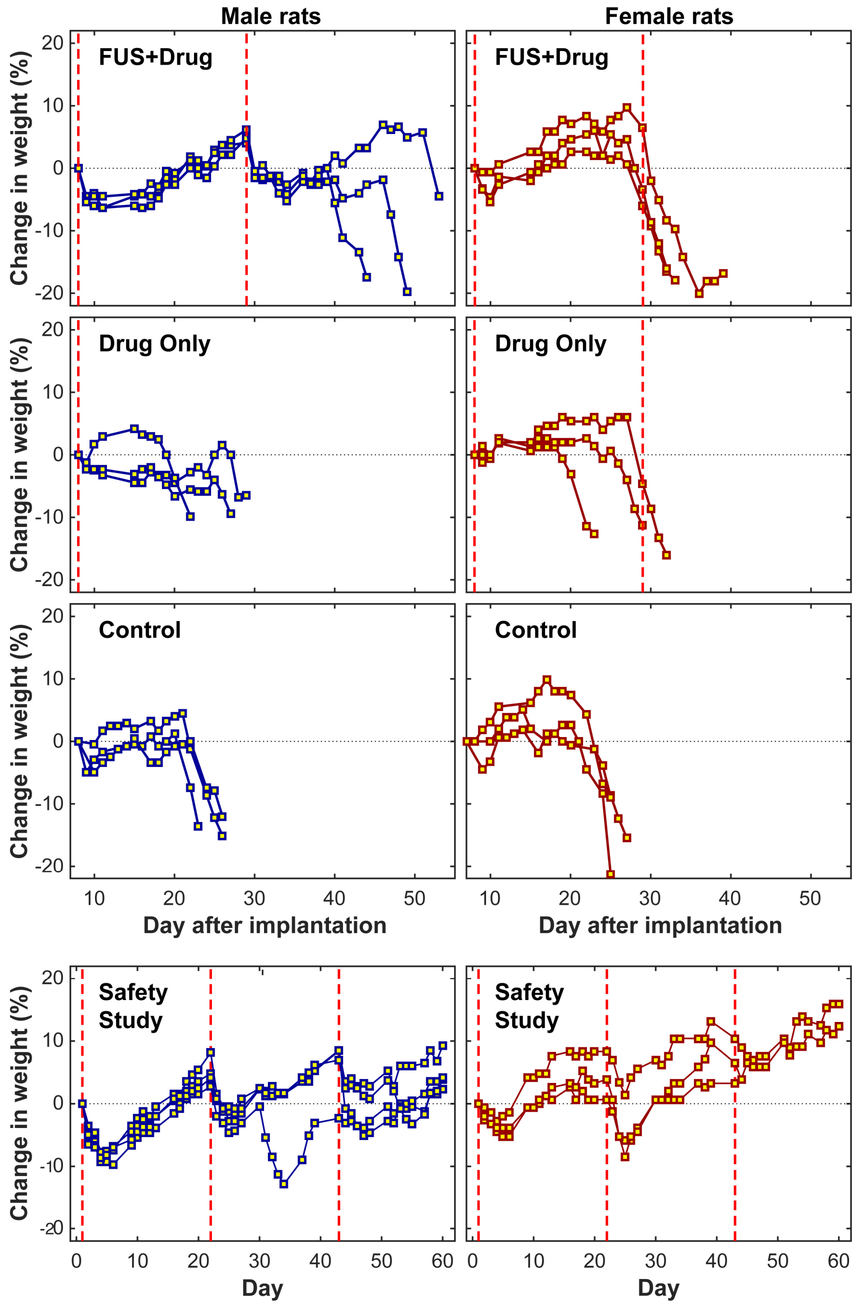

Figure S8: Change in weight in the rats during the efficacy and safety studies. The time of the treatments are noted by the vertical dotted lines. The male rats' weights were increasing at the time of the second FUS+carboplatin treatment while the females' weights were in decline. Weight loss apparently resulting from carboplatin was observed in many rats.

Table S1: Controller performance over 20 consecutive sessions of FUS-induced BBB disruption

		All	Loc. 1	Loc. 2
**Harmonic emission achieved controller goal (bursts)**	**No MB**	0.03% (2/7366)	0% (0/3684)	0.05% (2/3682)
	**MB**	30% (6702/22408)	30% (3335/11204	30% (3367/11204)
**Subharmonic emission (bursts)**	**No MB**	0.30% (23/7366)	0.30% (11/3684)	0.30% (12/3682)
	**MB**	0.10% (26/22408)	0.20% (17/11204)	0.08% (9/11204)
**Wideband emission (bursts)**	**No MB**	0% (0/7366)	0% (0/3684)	0% (0/3682)
	**MB**	0.10% (28/22408)	0.07% (8/11204)	0.20% (20/11204)
**Bursts at maximum power**	**MB**	8.4% (1881/22408)	14% (1531/11204	3.1% (350/11204)
**Harmonic emission achieved controller goal (locations)**	**No MB**	0.7% (2/306)	0% (0/153)	1.3% (2/153)
	**MB**	100% (305/306)	100% (153/153)	99% (152/153)
**Subharmonic emission (locations)**	**No MB**	5.6% (17/306)	5.2% (8/153)	5.9% (9/153)
	**MB**	7.5% (23/306)	9.8% (15/153)	5.2% (8/153)
**Wideband emission (locations)**	**No MB**	0% (0/306)	0% (0/153)	0% (0/153)
	**MB**	8.2% (25/306)	4.6% (7/153)	12% (18/153)
**Locations at maximum power**	**MB**	26% (80/306)	33% (50/153)	20% (30/153)

(MB: microbubbles)

Table S2: Measurements and outcomes for the sonications in the safety study

	All	Volume 1	Volume 2	P
**ΔR1 (s^{-1})**	0.061 ± 0.020	0.065 ± 0.020	0.058 ± 0.020	0.003
**Contrast enhancement (% increase)**	10.0 ± 4.3	10.6 ± 5.0	9.5 ± 3.6	0.03
**Acoustic energy (mJ)**	102.8 ± 23.8	112.7 ± 20.7	92.8 ± 22.5	<0.001
**Final acoustic power (W)[1]**	0.35 ± 0.09	0.38 ± 0.08	0.31 ± 0.09	<0.001
**Final pressure amplitude (kPa)[1]**	152 ± 21	161 ± 17	144 ± 20	<0.001
**Peak Harmonic emission (dB)**	11.9 ± 3.3	10.8 ± 2.5	13.1 ± 3.7	<0.001
**Integrated Harmonic emission (dB)**	428.4 ± 3.3	422.8 ± 2.5	434.0 ± 3.7	0.07

ΔR1 and contrast enhancement (% increase) are between sonicated and control hemispheres; emissions were normalized to noise floor

[1]Mean power and pressure amplitude between 25-75 s

Table S3: Intact carboplatin concentrations in tumor and normal brain

	Carboplatin conc. (μg/g)[a]				Carboplatin conc. tissue/plasma ratio[a]	
**Group**	**FUS**	**Control**	**Ratio**	**P[b]**	**FUS**	**Control**
**Tumor at 1h:**	21.55 (19.5)	7.43 (42.7)	2.90 (24.1)	0.016	0.80 (6.9)	0.28 (25.8)
**Brain at 1h:**	11.14 (34.1)	1.53 (10.9)	7.30 (26.2)	0.006	0.41 (10.8)	0.06 (18.7)
**Tumor at 4h:**	6.35 (11.3)	2.66 (16.2)	2.39 (25.0)	0.026	10.46 (10.2)	4.38 (28.6)
**Brain at 4h:**	5.08 (10.9)	1.21 (17.2)	4.20 (21.9)	0.007	8.36 (8.6)	1.99 (28.9)

[a] Results are presented as the geometric mean (geometric %CV); Plasma concentrations: 26.9 (23.9) and 0.61 (19.6) μg/ml at one and four hours, respectively.

[b] Paired two-tailed t-test of log transformed data
